# Association between hypertension and dietary inflammatory index among adults: a cross-sectional study in Hainan, China

**DOI:** 10.3389/fnut.2026.1793837

**Published:** 2026-07-15

**Authors:** Yan Fu, Diyue Liu, Yinuo Cheng, Yulu Xia, Xinxin Kuang, Sujuan Jiang, XinMei Lai, YongZhong Ma, Bin He

**Affiliations:** 1 Hainan Provincial Center for Disease Control and Prevention (Hainan Academy of Preventive Medicine), Haikou, China; 2 School of Nursing, Liaoning University of Traditional Chinese Medicine, Shenyang, China; 3 School of Public Health, Hainan Medical University, Haikou, China

**Keywords:** cross-sectional study, dietary inflammatory index, hypertension, resident, risk factors

## Abstract

**Background:**

Hypertension (HTN) affects 31.6% of Chinese adults and contributes to cardiovascular and cerebrovascular diseases through chronic inflammation. Pro-inflammatory diets, measured by the Dietary Inflammatory Index (DII), have been associated with increased HTN risk. However, evidence from tropical regions remains limited. As China’s only tropical province, Hainan has distinctive dietary patterns shaped by its geographic and climatic characteristics. This study aimed to examine the association between the DII and HTN among adults in Hainan to identify high-risk dietary patterns and inform targeted prevention strategies.

**Methods:**

A cross-sectional study was conducted between 2020 and 2022 using a multistage sampling design to assess dietary intake among adults in Hainan. Data included demographic characteristics, height and weight, blood pressure assessments, and three-day dietary recalls. Quantile regression models were applied to evaluate the association between the DII and HTN. Subgroup analyses explored potential effect.

**Results:**

A total of 826 adults were included, including 359 men (43.5%) and 467 women (56.5%). In unadjusted quantile regression, only new-onset HTN showed a significant inverse association with DII compared to normal blood pressure, confined to the median quantile (*β* = −0.96, 95% CI: −1.89 to −0.04; *p = 0.040*). However, this association was attenuated after adjustment for confounders and was no longer statistically significant (adjusted *p > 0.05*). Subgroup analyses revealed DII values were less likely to be at the median among female and Han Chinese with incident HTN, and among hypertensives aged 34–54 years, whereas in the 55–64 age group, those with incident HTN and no prior history were less likely to fall at the lower end of the DII distribution. No significant associations were found in males, ethnic minorities, or across occupational physical activity intensity categories.

**Conclusion:**

This study suggests a potential relationship between dietary inflammatory potential and blood pressure in Hainan adults. No significant association persisted after full adjustment; however, an association between DII and new-onset HTN was observed at the median quantile in unadjusted models. Consistent trends in subgroup explorations indicate that pro-inflammatory dietary patterns may be associated with elevated blood pressure in specific populations, such as females, Han Chinese, and adults aged 34–64 years.

## Introduction

According to the Chinese Guidelines for the Prevention and Treatment of Hypertension(HTN) (Revised 2024 edition), individuals with a systolic blood pressure (SBP) ≥ 140 mmHg and/or diastolic blood pressure (DBP) ≥ 90 mmHg, measured in the consulting room and without the use of antihypertensive medications, are classified as hypertensive (1). The 2023 China Cardiovascular Health Report indicates a HTN prevalence of 31.6% among adults, with 43.1% of normotensive individuals exhibiting high-normal blood pressure, a critical prehypertensive state (2). This elevated burden persists across all regions, as evidenced by consistent prevalence rates of 30 ~ 40% throughout eastern, central, and western China (3–5). As research on HTN advances, accumulating evidence underscores its role as a major risk factor for a wide range of serious health conditions. Additionally, hypertensive crises, resulting from uncontrolled HTN, are significant contributors to the reduction in patients’ healthy life expectancy (6).

The risk factors for HTN are complex and multifaceted, and their full scope remains incompletely understood. In 2007, researchers identified that immune cells play a causal role in both HTN and organ damage (7), leading to the hypothesis that chronic low-grade inflammation may contribute to the development of HTN and other related conditions (8). Studies have demonstrated that dietary modifications, particularly those focused on anti-inflammatory foods, can help mitigate inflammation associated with HTN, type 2 diabetes (T2DM), and other risk factors (9). In 2014, Shivappa et al. introduced the Dietary Inflammation Index (DII) as a tool to assess diet-induced inflammation (10). The DII quantifies the pro-inflammatory or anti-inflammatory potential of an individual’s diet by evaluating the inflammatory impact of each dietary component, which are then aggregated to provide an overall score. Cumulative evidence indicates a significant dose–response relationship between pro-inflammatory diets (quantified by DII) and the incidence of HTN (11). Mechanistically, diet-induced chronic low-grade inflammation, particularly through gut-mediated pathways, plays a crucial role in HTN pathogenesis (12, 13). Emerging evidence suggests that chronic low-grade inflammation plays a central role in the pathogenesis of HTN (14). The DII, a literature-derived tool that quantifies the inflammatory potential of the overall diet, has been widely applied to examine associations between dietary patterns and cardiometabolic outcomes (15, 16). Most previous studies have treated DII as an exposure variable when evaluating HTN risk, and findings generally indicate that pro-inflammatory diets are associated with increased odds of elevated blood pressure (17). However, limited research has examined the distribution of dietary inflammatory potential across different blood pressure categories, which may provide additional insight into dietary patterns among individuals at different stages of HTN.

Furthermore, existing evidence on the association between DII and HTN is predominantly derived from developed countries, with relatively few studies conducted in developing settings (11, 12). Data from tropical regions are particularly scarce. Hainan Province, the only tropical province in China, has unique geographic, climatic, and cultural characteristics that shape dietary patterns distinct from those of other regions. Given these contextual differences, it is important to determine whether the relationship between dietary inflammatory potential and blood pressure observed elsewhere is consistent in tropical populations. Therefore, this study aims to examine the association between blood pressure levels and DII scores among adults in Hainan. We hypothesize that dietary inflammatory potential differs across blood pressure categories. The findings may provide region-specific evidence to inform dietary strategies for HTN prevention and management in tropical developing regions.

## Materials and methods

### Study design

**Figure 1:**
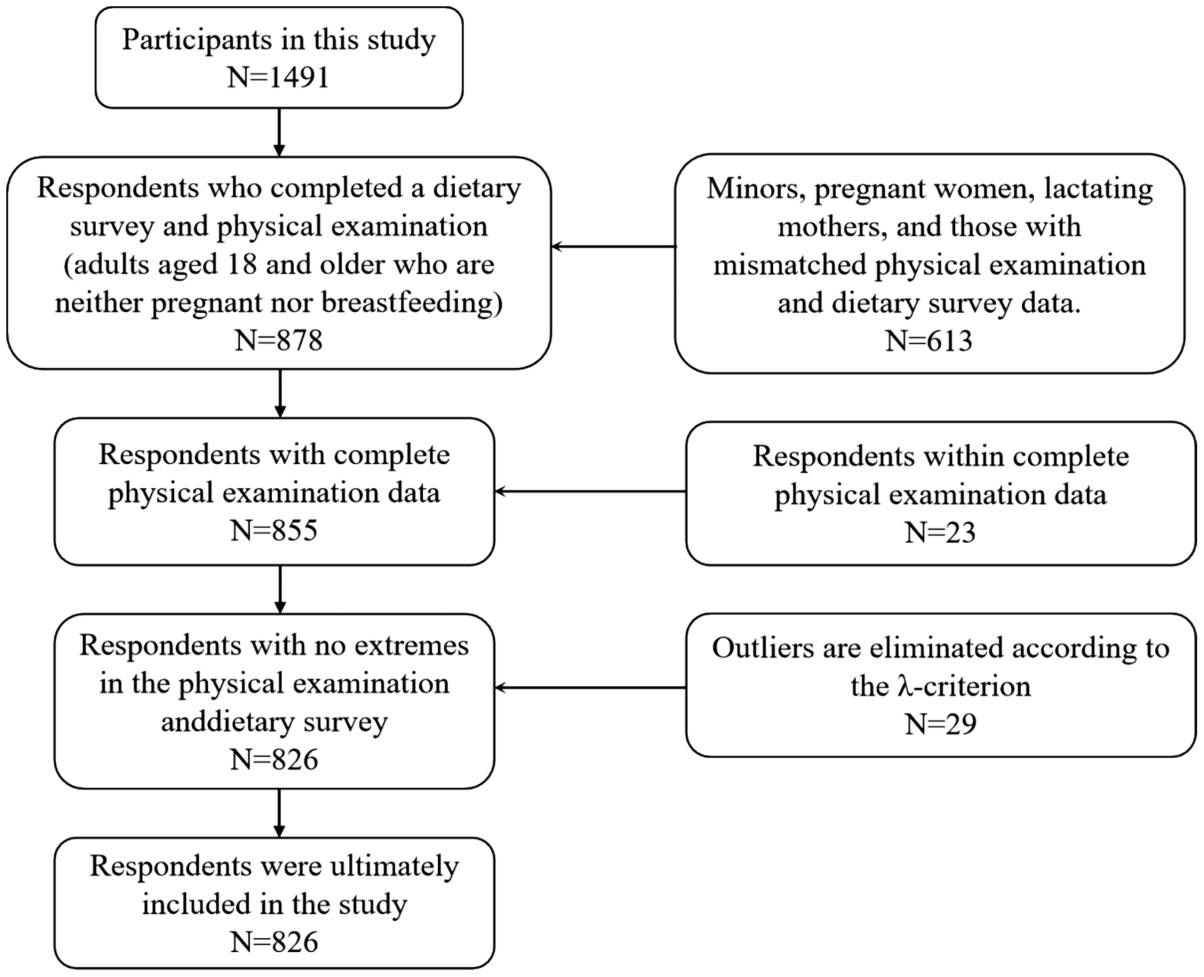
Flowchart of sample sizes.

This population-based cross-sectional study (2020–2022) employed a multi-stage sampling framework across Hainan Province to assess adult dietary habits. Using random number tables, we first selected six representative cities/counties. Subsequently, Probability Proportional to Size (PPS) sampling was applied to select two towns/streets per region, proceeding to random selection of one village/neighborhood committee per cluster. Within each community, we systematically recruited 25 consecutive households, enrolling all residents aged ≥18 years for dietary surveys and physical measurements. The protocol followed Chinese Center for Disease Control and Prevention’s nationally standardized 2015 Nutrition and Health Monitoring Program (Ethics No. 2022-008) in accordance with Declaration of Helsinki principles, with ethical approval from the Ethics Review Committee of Hainan Provincial Center for Disease Control and Prevention (approval No. 2024037) and documented informed consent from all participants. This study was conducted through a community-based survey of participants and did not involve the collection of any biological samples. Exclusion criteria: (1) Individuals under the age of 18; (2) Visitors who are not permanent or temporary adults of the selected households; (3) Individuals unable to read or independently complete the survey; (4) Individuals who have not signed the informed consent form. Inclusion criteria: (1) Age ≥ 18 years; (2) Provision of informed consent and willingness to participate; (3) Capacity to understand the survey content independently or with assistance from a trained interviewer. Out of an initial 1,491 adults enrolled, 826 qualified participants were retained after excluding those with incomplete questionnaires (missing data ≥ 20%) or invalid blood pressure measurements (Figure 1).

### Investigation contents and methods

#### Dietary survey

Dietary intake was assessed using a non-consecutive 3-day dietary recall, including one weekend day. This method has been validated in Chinese populations and is considered a reliable approach for assessing individual dietary intake (18). Detailed records were collected for all foods and beverages consumed by participants, including snacks, meals consumed outside the home, and any food provided to guests. Salt and condiment usage was quantified by weighing, and total intake over the 3-day period was calculated. Based on these comprehensive dietary records, the daily intake of various food components (nutrients) for each participant was calculated using the Chinese Food Composition Table (6th Edition) (19). Food types and quantities were converted into their corresponding nutrient and energy content according to this standard reference. The average daily nutrient intake for each participant was then determined and assessed relative to the Chinese Dietary Reference Intakes (2023 Edition) (20). Additionally, intake of beans and bean products was standardized to dried bean equivalents using a protein-based conversion ratio (35.1 g protein per 100 g dried beans).

#### Calculation of dietary inflammatory index

This study calculated the DII using the updated methodology by Shivappa et al. (10), or each participant, daily intake of 21 key food components [wine, caroten, kcal, protein, fat, fiber, Vitamin A, thiamin, riboflavin, Vitamin C, Vitamin E, mg, fe, zn, se, cholesterol, ginger(g), garlic(g), caffeine(g), onion(g), tea(g)] derived from 3-day dietary recalls was standardized against the global reference database to calculate Z-scores, representing deviations from global mean intakes. To normalize distribution skewness, these Z-scores were converted to percentiles and then transformed to a symmetric scale ranging from −1 (maximally anti-inflammatory) to +1 (maximally pro-inflammatory). Each transformed value was weighted by its literature-derived inflammatory effect score (10), and the weighted values for all 21 components were summed to generate a daily DII score. The mean of three daily scores per participant served as the final DII value, representing habitual dietary inflammatory potential. In this study, DII is treated as a continuous variable and is calculated using its percentiles.

#### Diagnostic methods for hypertension

In this study, blood pressure was measured three times, with a 1-min interval between each measurement. The average of the last two measurements was used as the participant’s blood pressure result. HTN was defined as having a mean SBP ≥ 140 mmHg (18.6 kPa) and/or a mean DBP ≥ 90 mmHg (12 kPa). Elevated blood pressure (normal high value) was defined as mean SBP ≥ 120 mmHg and <140 mmHg, or mean DBP ≥ 80 mmHg and <90 mmHg.

#### Body measurements

Participants should remove shoes, coats, and other objects during height and weight measurements. They should stand with their back to the SGZ-210 column height meter, keeping their torso straight, eyes forward, arms by their sides, legs straight, heels together, and toes at a 60° angle. The heel, sacrum, and area between the scapulae should align with the column. Height and weight are measured using the SGZ-210 column height meter and TC-200K electronic weighing scale. Height is recorded in centimeters (cm) and weight in kilograms (kg), both to one decimal place, with measurement errors not exceeding 0.5 units.

#### Blood pressure measurement

Blood pressure was measured using the Omron HBP-1300 electronic sphygmomanometer in a quiet, warm, and private room. Participants were instructed to avoid strenuous exercise, eating, or caffeine for at least 1 hour before measurement, refrain from smoking for 30 min prior, and rest for 15 min. Measurements were performed in the morning, preferably on the right upper arm (left arm if necessary), with the cuff placed on bare skin 1–2 cm above the elbow. Participants were asked to sit quietly for 5 min before the measurement.

In addition to the measured values, participants were asked whether they had been previously diagnosed with HTN. Based on both measured blood pressure and self-reported history, participants were classified into the following categories: ①Normal; ②High-normal (with history of HTN); ③High-normal (without history of HTN); ④New-onset HTN; ⑤HTN.

#### Questionnaire: general demographic information

The questionnaire includes basic demographic details such as nation, gender, date of birth, and occupational physical activity intensity.

Occupational physical activity intensity was assessed through a single self-administered structured questionnaire item. Participants classified the intensity of their usual work-related physical activity into one of three predefined categories (e.g., light, moderate, heavy) based on standardized criteria and illustrative examples provided.

Occupational physical activity intensity was categorized as:

1. *Light*: approximately 75% of working time spent sitting or standing and 25% engaged in occupational activities, such as office work, appliance repair, retail sales, hotel service, laboratory procedures, and teaching;
2. *Moderate*: approximately 25% of working time spent sitting or standing and 75% engaged in occupational activities, such as routine student activities, motor vehicle driving, electrical installation, lathe operation, and metal cutting;
3. *Heavy*: approximately 40% of working time spent sitting or standing and 60% engaged in physically demanding activities, including non-mechanized agricultural labor, steel production, dancing, sports activities, loading and unloading, and mining.

Detailed descriptions and occupational examples were provided within the questionnaire to assist participants in accurately determining their occupational physical activity intensity category. In addition, trained survey staff were available on-site to provide clarification when necessary, ensuring consistent understanding of the classification criteria.

### Statistical analysis

This study utilized R software 4.3.3 (R Foundation for Statistical Computing, Vienna, Austria) for data analysis. Initially, qualitative data were described using frequencies and percentages (*N*%). Subsequently, the Shapiro–Wilk test was employed to assess normality; non-normally distributed data were represented using quartiles, and inter-group comparisons were conducted using the Kruskal–Wallis test. Additionally, univariate kernel density estimation was used to plot density curves for each variable, providing a visual representation of the data distribution. Finally, given the non-normal distribution of the data, quantile regression was employed to examine associations between independent variables and the DII. The model was adjusted using the variables gender, age, nation, and occupational physical activity intensity. Subgroup analyses were performed to assess differences in outcomes across various characteristic groups to further explore potential influencing factors. All statistical analyses were conducted using two-sided tests, with a significance level set at *α* = 0.05.

## Result

### Demographic characteristics of respondents

Of the 826 participants, 56.5% were female and 43.5% male. The predominant age group was 35–54 years (37.2%), whereas those aged 18–34 years constituted only 12.6%. Han nationality was the most frequent ethnicity (68.4%), whereas minority nationalities comprised 31.6%. Light occupational physical activity intensity was reported by 51.1% of participants, moderate by 37.8%, and heavy by 11.1%. High-normal blood pressure (with history of HTN) was the leading category (44.9%). The DII was divided into six quantiles, and the representation across these quantiles was roughly uniform by gender, age, nationality, occupational physical activity intensity, and blood pressure status. For further details, see Table 1.

**Table 1:** Demographic variables and dietary inflammation index by quintile.

Variable	*N*	DII Quantile					
		<0.10	[0.10, 0.25)	[0.25 ~ 0.50)	[0.50 ~ 0.75)	[0.75 ~ 0.90)	>0.90
Gender							
Male	359 (43.5%)	37 (10.31%)	52 (14.48%)	78 (21.73%)	87 (24.24%)	63 (17.55%)	42 (11.70%)
Female	467 (56.5%)	47 (10.06%)	70 (14.99%)	130 (27.84%)	119 (25.48%)	61 (13.06%)	40 (8.57%)
Age (years)							
18~	104 (12.6%)	13 (12.50%)	19 (18.27%)	24 (23.08%)	27 (25.96%)	9 (8.65%)	12 (11.54%)
35~	307 (37.2%)	21 (6.84%)	45 (14.66%)	73 (23.78%)	72 (23.45%)	55 (17.92%)	41 (13.36%)
55~	206 (24.3%)	18 (8.74%)	24 (11.65%)	57 (27.67%)	62 (30.10%)	29 (14.08%)	16 (7.77%)
65~	209 (24.7%)	31 (14.83%)	34 (16.27%)	54 (25.84%)	45 (21.53%)	31 (14.83%)	13 (6.22%)
Nation							
Han	565 (68.4%)	62 (10.97%)	84 (14.87%)	148 (26.19%)	156 (27.61%)	81 (14.34%)	34 (6.02%)
Minority^a^	261 (31.6%)	22 (8.43%)	38 (14.56%)	60 (22.99%)	50 (19.16%)	43 (16.48%)	48 (18.39%)
Occupational physical activity intensity							
Light	422 (51.1%)	44 (10.43%)	72 (17.06%)	112 (26.54%)	119 (28.20%)	55 (13.03%)	20 (4.74%)
Moderate	312 (37.8%)	29 (9.29%)	36 (11.54%)	78 (25.00%)	68 (21.79%)	54 (17.31%)	47 (15.06%)
Heavy	92 (11.1%)	11 (11.96%)	14 (15.22%)	18 (19.57%)	19 (20.65%)	15 (16.30%)	15 (16.30%)
Blood pressure level							
Normal	169 (19.6%)	14 (8.28%)	26 (15.38%)	36 (21.30%)	50 (29.59%)	27 (15.98%)	16 (9.47%)
High-normal (with history of HTN)	371 (44.9%)	36 (9.70%)	52 (14.02%)	92 (24.80%)	96 (25.88%)	58 (15.63%)	37 (9.97%)
High-normal (without history of HTN)	55 (6.7%)	9 (16.36%)	5 (9.09%)	13 (23.64%)	18 (32.73%)	9 (16.36%)	1 (1.82%)
New-Onset HTN	138 (16.7%)	15 (10.87%)	27 (19.57%)	36 (26.09%)	22 (15.94%)	19 (13.77%)	19 (13.77%)
HTN	93 (11.3%)	10 (10.75%)	12 (12.90%)	31 (33.33%)	20 (21.51%)	11 (11.83%)	9 (9.68%)

a The minority groups included the Li, Miao, Yao, Korean, and Bouyei.

### Dietary nutrient intake among respondents with varying blood pressure levels

In this study, we compared nutrient intake among respondents with different blood pressure levels. There were no statistically significant differences in energy, protein, dietary fiber, vitamin A, vitamin B_1_, vitamin B_2_, niacin, vitamin C, or sodium intake among the groups (all *p* > 0.05).

Significant differences were observed for fat intake (H = 11.63, *p* < 0.05), retinol (H = 9.70, *p* < 0.05), cholesterol (H = 9.59, *p* < 0.05), and Body Mass Index (BMI) (H = 33.88, *p* < 0.001). Median fat intake was highest in the normal blood pressure group and lowest in the high-normal (without history of HTN) group, with Bonferroni-adjusted pairwise differences indicated by distinct superscript letters. Retinol intake was highest in the high-normal (without history of HTN) group and lowest in the HTN group. Cholesterol intake was highest in the high-normal (with history of HTN) group and lowest in the new-onset HTN group.

BMI increased across blood pressure categories, with the highest median BMI observed in the high-normal (without history of HTN) group and the lowest in the normal blood pressure group; pairwise comparisons showed statistically significant differences between groups as indicated by superscript letter. (For more details, see Table 2).

**Table 2:** Dietary nutrient intake of respondents [M(P_25_, P_75_)].

Variable	Blood pressure level					
	Normal	High-normal (with history of HTN)	High-normal (without history of HTN)	New-Onset HTN	HTN	*H*
Energy (kcal)	2179.72 (1841.82, 2813.20)	1791.78 (2193.59, 1791.78)	2086.35 (1656.50, 2471.15)	2120.69 (1808.72, 2877.20)	2186.02 (1769.50, 2714.01)	3.52
Protein(g)	92.25 (72.17, 124.84)	89.65 (71.84, 123.00)	84.61 (70.30, 113.76)	87.28 (71.90, 118.07)	87.35 (70.49, 120.00)	1.61
Fat(g)	104.20 (70.17, 136.52)^a^	97.64 (72.24, 134.89)	82.80 (65.77, 118.25)	91.36 (69.90, 125.89)	88.65 (68.03, 107.32)^b^	**11.63** ^*^
Fiber(g)	6.21 (3.73, 10.21)	6.16 (3.76, 10.88)	6.64 (3.74, 10.18)	6.05 (3.56, 9.10)	5.57 (3.28, 9.68)	1.59
Vitamin A (ugRAE)	422.57 (294.33, 670.93)	473.04 (277.33, 725.49)	393.07 (310.45, 571.15)	432.43 (268.18, 646.07)	396.50 (275.30, 576.66)	3.51
Retinol	130.83 (61.00, 223.60)	150.33 (76.90, 273.36)	154.33 (67.12, 224.53)	107.17 (64.29, 218.74)	102.53 (53.17, 201.03)	**9.70** ^*^
Vitamin B_1_ (mg)	1.12 (0.83, 1.48)	1.08 (0.83, 1.47)	1.03 (0.73, 1.52)	1.09 (0.81, 1.58)	1.09 (0.81, 1.54)	1.37
Vitamin B_2_ (mg)	0.97 (0.73, 1.34)	0.97 (0.72, 1.38)	0.93 (0.76, 1.14)	0.86 (0.67, 1.28)	0.88 (0.70, 1.17)	5.86
Niacin (mg)	22.78 (16.56, 30.72)	21.37 (15.89, 31.03)	19.74 (13.44, 25.62)	20.14 (14.88, 31.78)	20.52 (15.07, 28.5)	5.88
Vitamin C (mg)	108.98 (57.60, 183.84)	111.87 (56.88, 181.74)	106.67 (41.65, 162.50)	119.07 (53.61, 188.97)	105.92 (72.67, 174.53)	2.78
Na (mg)	4244.92 (2926.68, 6451.95)	4425.88 (2963.42, 7205.35)	3748.57 (2624.14, 5312.18)	4853.19 (3274.98, 7977.93)	4347.07 (2840.81, 7719.56)	8.31
Cholesterol (mg)	500.53 (315.40, 834.51)	580.33 (329.62, 854.50)	473.20 (332.07, 748.08)	469.26 (259.86, 736.45)	485.21 (279.47, 684.70)	**9.59** ^*^
BMI^a^	22.53 (20.25, 24.64)^a^	23.56 (21.49, 25.89)^b^	24.75 (22.96, 26.5)^c^	24.29 (21.92, 26.87)^d^	24.33 (21.64, 26.76)^e^	**33.88** ^***^

^a^Body Mass Index, BMI; *p < 0.050, ***p < 0.001.

a, b indicate two-by-two comparisons of student frequencies for different grades with the same nutritional status. “a vs. b,” “a vs. a, b” and “b vs. a, b” indicate statistically significant differences (Bonferroni correction, *p* < 0.017). “a vs. a” and “b vs. b” indicate that the differences are not statistically significant (Bonferroni correction, *p* > 0.017).

### Univariate kernel density estimation of DII distribution by subgroup

**Figure 2:**
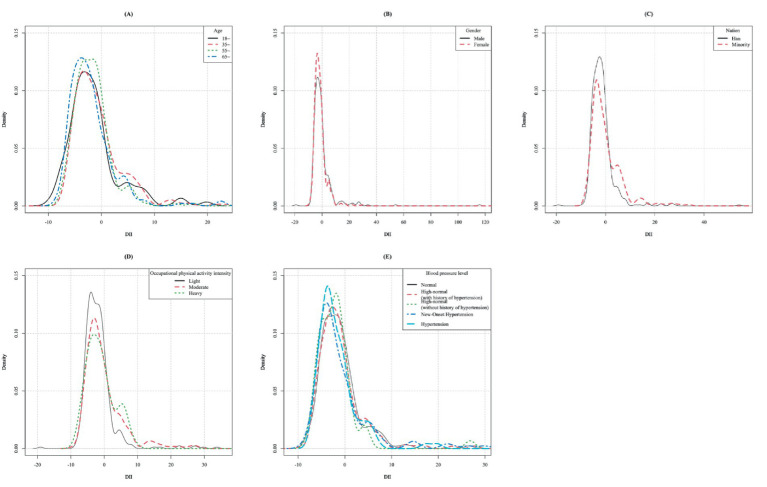
Kernel density curves of dietary inflammation index across different subgroups of respondents. **(A)** Displays the kernel density curves of DII for different age groups; **(B)** Compares the kernel density curves of DII for males and females. **(C)** Shows the kernel density estimates of DII for Han and Minority groups. **(D)** Presents the kernel density curves of DII for different Occupational physical activity intensity levels. **(E)** Displays the kernel density curves of DII for different blood pressure categories.

In this study, we performed univariate kernel density estimation to examine the distribution of the DII across different subgroups. The results revealed a pronounced skew in the distribution for all age groups, centered around a mean value of 5.14. Gender and ethnicity groups displayed areas of low frequency, suggesting potential imbalances in age distribution. The occupational physical activity intensity groups exhibited a multimodal distribution, reflecting individual variation across different levels of occupational physical activity intensity. Meanwhile, the blood pressure categories showed a more concentrated distribution, with values ranging from a minimum of −11.39 to a maximum of 29.64. For further details, see Figure 2.

### Differences in blood pressure levels on dietary inflammatory indices: quantile regression analysis

In the crude model, compared with participants with normal blood pressure, those with new-onset HTN were less likely to exhibit DII values at the median of the distribution (*τ* = 0.50; *β* = −0.96, 95% CI: −1.89 to −0.04, *p = 0.040*). However, after adjustment for gender, age, nation, and occupational physical activity intensity, blood pressure status was no longer associated with the likelihood of being within any specific quantile range of the DII distribution. Regarding covariates, compared with males, females were more unlikely to exhibit DII values at or below the median (*τ* = 0.50; *β* = −0.53, *p = 0.013*). Compared with participants aged 18–34 years, those aged 35–54 and 55–64 years were more likely to present with higher DII values at the lower quantile (τ = 0.10). Compared with Han participants, ethnic minorities were more likely to exhibit DII values within the upper quantile range (τ = 0.75 and τ = 0.90). Similarly, compared with participants performing light-intensity occupational physical activity, those performing moderate-intensity occupational physical activity were more likely to have DII values in the upper end of the distribution (full details: Table 3; Supplementary Table S1).

**Table 3:** Quantile regression model of different blood pressure levels and dietary inflammation index.

Variable	DII Quantile 0.10		DII Quantile 0.25		DII Quantile 0.50		DII Quantile 0.75		DII Quantile 0.90	
Blood pressure level	β (95% CI)	*p*	β (95% CI)	*p*	β (95% CI)	*p*	β (95% CI)	*p*	β (95% CI)	*p*
Normal	Ref.	Ref.	Ref.	Ref.	Ref.	Ref.	Ref.	Ref.	Ref.	Ref.
High-normal (with history of HTN)	−0.22 (−0.85, 0.42)	0.505	0.07 (−0.61, 0.74)	0.845	−0.19 (−1.30, 0.92)	0.687	0.15 (−0.91, 1.20)	0.785	0.02 (−2.61, 2.65)	0.987
High-normal (without history of HTN)	−0.34 (−1.14, 0.46)	0.408	−0.71 (1.92, 0.49)	0.247	−0.19 (−1.30, 0.92)	0.741	−1.10 (−2.43, 0.23)	0.106	−3.33 (−7.08, 0.42)	0.082
New-Onset HTN	−0.38 (−1.14, 0.38)	0.326	−0.51 (−1.20, 0.19)	0.152	**−0.96** **(−1.89, −0.04)**	**0.040**	0.54 (−0.89, 1.98)	0.457	1.12 (−3.30, 5.53)	0.620
HTN	−0.37 (−1.22, 0.48)	0.393	0.03 (−0.73, 0.79)	0.941	−0.75 (−1.84, 0.33)	0.173	−0.36 (−1.65, 0.92)	0.579	−1.21 (−4.80, 2.38)	0.508
Blood pressure level	β_adj_ (95% CI)	*p*	β_adj_ (95% CI)	*p*	β_adj_ (95% CI)	*p*	β_adj_ (95% CI)	*p*	β_adj_ (95% CI)	*p*
Normal	Ref.	Ref.	Ref.	Ref.	Ref.	Ref.	Ref.	Ref.	Ref.	Ref.
High-normal (with history of HTN)	0.00 (−0.51, 0.62)	0.997	0.09 (−0.58, 0.76)	0.793	−0.32 (−1.16, 0.53)	0.465	0.03 (−1.06, 1.13)	0.955	−0.30 (−2.05, 1.45)	0.733
High-normal (without history of HTN)	−0.05 (−0.74, 0.64)	0.890	−0.05 (−0.78, 0.67)	0.888	−0.22 (−1.05, 0.60)	0.596	0.34 (−0.79, 1.46)	0.558	0.21 (−1.55, 1.97)	0.818
New-Onset HTN	−0.67 (−1.65, 0.31)	0.179	−1.07 (−2.76, 0.62)	0.241	−0.26 (−1.57, 1.05)	0.699	−0.87 (−2.53, 0.79)	0.303	−0.10 (−4.33, 4.13)	0.962
HTN	−0.13 (−0.89, 0.64)	0.748	−0.52 (−1.40, 0.36)	0.250	−0.91 (−1.95, 0.14)	0.089	0.27 (−1.38, 1.92)	0.747	0.34 (−3.58, 4.27)	0.865

“*β*” denotes the effect size estimated from the unadjusted model, whereas “β_adj_” denotes the effect size obtained from the adjusted model. Boldface indicates statistical significance.

In the subgroup analysis, associations between DII quantiles and blood pressure levels were explored across various demographic and lifestyle factors. Among females with new-onset HTN, DII values at the median quantile were significantly lower compared with normotensive females (*τ* = 0.50; *β* = −1.38, 95% CI: −2.56 to −0.20; *p* = 0.022), while no significant differences were observed among males across any blood pressure category. Regarding age subgroups, participants aged 35–54 years with HTN showed significantly lower DII values at the median quantile compared with normotensive peers (*τ* = 0.50; *β* = −2.54, 95% CI: −4.76 to −0.31; *p* = 0.026), and participants aged 55–64 years with new-onset HTN had lower DII values at the lower quantile (τ = 0.25; *β* = −2.12, 95% CI: −4.07 to −0.17; *p* = 0.034). For ethnicity, among Han participants, those with new-onset HTN were less likely to have DII values at the median quantile compared with normotensive Han participants (τ = 0.50; *β* = −1.28, 95% CI: −2.22 to −0.34; *p* = 0.008), whereas no significant associations were observed among ethnic minorities. In terms of occupational physical activity intensity, none of the associations reached statistical significance across blood pressure categories as detailed in Supplementary Table S2.

## Discussion

This study revealed significant differences in the intake of fat, retinol, vitamin B_2_, and cholesterol, as well as in BMI, across different blood pressure levels among Hainan adults. In unadjusted models, participants with new-onset HTN were less likely to have DII values at the median, whereas adjusted models suggested that DII distributions varied by gender, age, ethnicity, and occupational physical activity level.

Individuals with normal blood pressure had the highest energy intake, while those with high-normal blood pressure (without a history of HTN) had the highest retinol intake. The highest cholesterol intake was observed in individuals with high-normal blood pressure (with a history of HTN). This study focused on Hainan adults, whose island geography influences unique dietary patterns, including high seafood consumption, a primary source of animal fats. Our finding of higher fat intake among individuals with normal blood pressure aligns with Liu et al. (21), who suggested that marine-derived fats may help protect against blood pressure issues. Interestingly, retinol (vitamin A) intake was highest in the high-normal blood pressure group, while the lowest intake was observed in the hypertensive group. This is consistent with previous longitudinal studies, which have shown an association between serum retinol levels and both total mortality and cardiovascular mortality, suggesting that serum retinol may influence cardiovascular disease (CVD) mortality by modulating blood pressure (22). In contrast to prior research, the present study did not find a direct increase in blood pressure levels with higher cholesterol intake (23). Rather, the highest intake of cholesterol was observed in individuals with elevated blood pressure values. This could reflect that, where individuals with HTN may consciously adjust their diet to monitor cholesterol intake, potentially influencing the dietary reporting.

The findings of this study differ from those of Strath et al., who did not observe gender differences in the DII (24). In contrast, our study identified that females are more sensitive to the adverse health outcomes associated with changes in DII. The gender differences in DII observed in this study may be related to regional sociocultural factors. Hainan Province, with its unique traditional cultural system that evolved from a matrilineal society, exhibits a “female labor, male leisure” phenomenon, where women bear a greater burden of labor in society and the family (25). The more leisurely lifestyle of Hainanese men has given rise to the “Lao Ba Cha” (literally “old dad tea”) culture, providing local men with more time and opportunity to consume larger quantities of tea (26). Tea, being a highly anti-inflammatory component in the calculation of DII, may contribute to this difference. Additionally, there may be disparities in health literacy between genders, affecting their ability to access and apply health information. According to the research by Hao et al. (27), digital literacy and health literacy overlap in influencing the digital health behaviors of rural adults, with differences observed between surveyed males and females. This may explain the gender differences in participation in digital health behaviors and dietary choices. Males, due to their greater openness to innovation and pursuit of convenience, may be more inclined to accept and utilize digital health services, which could lead them to adopt diets with lower DII values after receiving accurate health information (27).

Compared to a larger-scale survey conducted in the United States, this study observed differences in the DII among respondents of different age groups, a phenomenon not noted in the American study (12). In this study, individuals aged 18 to 34, mostly in the early stages of university or career, often have to rely on ultra-processed foods as their main daily food source due to convenience and cost, despite their reluctance (28, 29). Ultra-processed foods have not only been indicated in previous research to lead to adverse health outcomes but are also considered to have a higher pro-inflammatory potential (30, 31). The variation in DII scores across age groups may be related to lifestyle choices. Individuals aged 55 to 64 years are either approaching retirement or have already retired and are capable of self-care. Similar to the conclusions of this study, evidence from China suggests that retirement can have a positive impact on the health of working-age populations within a certain range (32). Compared to younger groups, the increase in leisure time, coupled with anxiety about aging and personal health, may lead this cohort to choose a healthier, more anti-inflammatory dietary pattern (33). Compared to older groups, this population has better economic status and stronger self-care abilities, with both subjective and objective conditions to more actively adopt a healthy dietary pattern.

A study conducted in the United States found that the association between dietary patterns and the DII varies across different racial/ethnic groups, which is similar to our observation of differences in DII between Han and minority ethnic groups (34). The differences in DII between Han and minority ethnic groups in this study may be related to dietary structure. Such differences may be due to traditional dietary habits and cultural differences among different ethnicities. The indigenous Li ethnic group of Hainan is often characterized in official reports as a high-frequency user of tobacco and alcohol, and these lifestyle habits could potentially contribute to the observed disparities in the DII (35). Regarding occupational physical activity intensity, the conclusions of this study differ from previous research. Compared to heavy-intensity occupational physical activity laborers, light-intensity occupational physical activity laborers are less likely to have a pro-inflammatory diet. However, in the study by Byrd et al. (36), it appears that respondents with fewer physical activity sessions have a greater potential for pro-inflammatory diets. The differences in conclusions are likely due to the different definitions of occupational physical activity intensity in previous studies compared to the occupational physical activity intensity in this study. However, according to the International Society of Sports Nutrition, adequate protein intake after physical activity (e.g., resistance exercise) is necessary to meet individual needs (37). In this study, protein is a pro-inflammatory substance, so the reason why heavy-intensity occupational physical activity laborers have a more pro-inflammatory dietary pattern may be due to the increased demand for protein intake after heavy-intensity occupational physical activity.

Previous studies have shown a significant link between HTN, as well as other CVD and DII. Zhou et al. (12) found that hypertensive individuals had higher DII scores, indicating that respondents with higher blood pressure are likely to have higher levels of inflammatory factors in their dietary behaviors. Similarly, a study conducted in China revealed that individuals with HTN consumed more alcohol than those without HTN, and alcohol intake can substantially increase the levels of inflammatory factors in their diet (38). The crude models in this study clarified the HTN-DII relationship, consistent with previous studies. Dong et al. (39) suggested that cultural factors across regions may affect this association, and longitudinal studies have shown that the link between healthy eating indices and HTN varies by gender and race (40). Therefore, model adjustments were essential for this study.

In the present study, we observed that respondents with elevated blood pressure levels were more likely to exhibit significant differences in the DII compared to those with normal blood pressure. This suggests that the dietary patterns of individuals with high blood pressure tend to be more pro-inflammatory. Individuals with elevated blood pressure are considered a potential group at risk for HTN, and preventing the transition from elevated blood pressure to HTN holds significant public health implications (41, 42).

### Limitations and strengths

This study has several limitations. First, its cross-sectional design allows for the identification of associations but does not establish causal relationships between the DII and blood pressure. Second, while we acknowledge the potential for residual confounding, key confounders such as smoking, alcohol consumption, family history of HTN, and socioeconomic factors were not systematically collected due to operational constraints during fieldwork. These variables were not included in the analysis as the primary focus of this study was on dietary intake, particularly food consumption and seasoning measurements, within the limited time and budget available for the fieldwork.

In addition to the above-mentioned limitations, this study still has significant strengths. Conducted in Hainan’s representative tropical marine climate, this provides crucial baseline data for similar-latitude regions, enabling cross-climate diet-blood pressure comparisons. Beyond single-nutrient analysis, it comprehensively examines multi-nutrient (fat, retinol, vitamin B₂, cholesterol) and BMI relationships with blood pressure. Precise three-tier blood pressure stratification (normotensive/high-normal/hypertensive) – with particular focus on the clinically critical high-normal group – enables targeted dietary risk factor identification, offering significant public health implications.

## Conclusion

This study identified a significant association between the blood pressure levels and DII among adults of Hainan Province. Compared with normotensive individuals, those with incident HTN were less likely to have their DII in the middle tertile. This association was moderated by demographic characteristics, with effect modification observed across sex, age, ethnicity, and occupational physical activity level. The relationship between DII and blood pressure differed among females, Han, and adults (aged 35–64).

These findings suggest a link between dietary inflammation and blood pressure status. The observed population-specific patterns may inform future research, particularly in designing longitudinal or intervention studies aimed at establishing whether dietary modifications to lower DII can contribute to HTN prevention in high-risk groups.

## Data Availability

The datasets presented in this article are not readily available because they contain sensitive information that is subject to strict respondent privacy protection and government data security regulations. Requests to access the datasets should be directed to Diyue Liu, L15917189871@outlook.com.
