# Supplementary material for: Association between hypertension and dietary inflammatory index among adults: a cross-sectional study in Hainan, China
Source: Front Nutr. 2026 Jul 15;13:1793837. doi: 10.3389/fnut.2026.1793837 (PMC13418119; doi:10.3389/fnut.2026.1793837)
Supplement: Supplementary file 1 [file Table_1.DOCX]

| Table S1 Quantile regression model of different blood pressure levels and Dietary Inflammation Index(adjust) | | | | | | | | | | | |
| --- | --- | --- | --- | --- | --- | --- | --- | --- | --- | --- | --- |
| Variable | | DII Quantile 0.1 | | DII Quantile 0.25 | | DII Quantile 0.50 | | DII Quantile 0.75 | | DII Quantile 0.9 | |
|  |  | β（95%CI） | P | β（95%CI） | P | β（95%CI） | P | β（95%CI） | P | β（95%CI） | P |
| Gander | |  |  |  |  |  |  |  |  |  |  |
|  | Male | Ref. | Ref. | Ref. | Ref. | Ref. | Ref. | Ref. | Ref. | Ref. | Ref. |
|  | Female | 0.04(-0.45,0.52) | 0.882 | -0.14(-0.74,0.45) | 0.640 | **-0.53(-1.23,0.16)** | **0.013** | -0.67(-1.46,0.12) | 0.098 | -1.62(-3.49,0.24) | 0.088 |
| Age(years) | |  |  |  |  |  |  |  |  |  |  |
|  | 18~ | Ref. | Ref. | Ref. | Ref. | Ref. | Ref. | Ref. | Ref. | Ref. | Ref. |
|  | 35~ | **1.43(0.174,2.69)** | **0.026** | 0.65(-0.21,1.50) | 0.137 | 0.86(-0.20,1.92) | 0.114 | 0.91(-0.34,2.16) | 0.156 | 0.56(-2.78,3.91) | 0.742 |
|  | 55~ | **1.41(0.10,2.72)** | **0.035** | 0.66(-0.32,1.64) | 0.185 | 0.75(-0.40,1.89) | 0.201 | 0.26(-0.86,1.39) | 0.645 | -0.52(-4.04,3.00) | 0.774 |
|  | 65~ | 0.64(-0.60.1.88) | 0.311 | 0.03(-0.91,0.98) | 0.947 | -0.03(-1.18,1.12) | 0.959 | -0.08(-1.46,1.29) | 0.906 | 0.45(-3.42,4.32) | 0.819 |
| Nation | |  |  |  |  |  |  |  |  |  |  |
|  | Han | Ref. | Ref. | Ref. | Ref. | Ref. | Ref. | Ref. | Ref. | Ref. | Ref. |
|  | Minority | 0.27(-0.45,0.98) | 0.469 | -0.04(-0.58,0.51) | 0.891 | -0.10(-0.97,0.76) | 0.815 | **1.76(0.17,3.35)** | **0.030** | **3.39(0.856,5.93)** | **0.009** |
| Occupational physical activity intensity | |  |  |  |  |  |  |  |  |  |  |
|  | Light | Ref. | Ref. | Ref. | Ref. | Ref. | Ref. | Ref. | Ref. | Ref. | Ref. |
|  | Moderate | 0.48(-0.08,1.04) | 0.093 | 0.30(-0.32,0.92) | 0.341 | 0.60(-0.10,1.31) | 0.094 | **1.80(0.85,2.74)** | **<0.001** | **3.30(0.91,5.69)** | **0.007** |
|  | Heavy | -0.08(-1.11,0.96) | 0.882 | -0.14(-1.10,0.82) | 0.772 | 0.81(-0.45,2.08) | 0.207 | 1.29(-0.65,3.23) | 0.193 | 1.34(-1.11,3.79) | 0.283 |
| Blood pressure level | |  |  |  |  |  |  |  |  |  |  |
|  | Normal | Ref. | Ref. | Ref. | Ref. | Ref. | Ref. | Ref. | Ref. | Ref. | Ref. |
|  | High-normal  (with history of HTN) | -0.05(-0.74,0.64) | 0.890 | -0.05(-0.78,0.67) | 0.888 | -0.22(-1.05,0.60) | 0.596 | 0.34(-0.79,1.46) | 0.558 | 0.21(-1.55,1.97) | 0.818 |
|  | High-normal  (without history of HTN) | -0.67(-1.65,0.31) | 0.179 | -1.07(-2.76,0.62) | 0.241 | -0.26(-1.57,1.05) | 0.699 | -0.87(-2.53,0.79) | 0.303 | -0.10(-4.33,4.13) | 0.962 |
|  | New-Onset HTN | -0.13(-0.89,0.64) | 0.748 | -0.52(-1.40,0.36) | 0.250 | -0.91(-1.95,0.14) | 0.089 | 0.27(-1.38,1.92) | 0.747 | 0.34(-3.58,4.27) | 0.865 |
|  | HTN | 0.14(-0.95,1.23) | 0.800 | -0.13(-0.956,0.69) | 0.750 | -0.47(-1.66.723) | 0.442 | 0.46(-1.27.2.20) | 0.601 | 0.53(-2.18,3.24) | 0.700 |

| Table S2 Subgroups of quantile regression model of different blood pressure levels and Dietary Inflammation Index | | | | | | | | |
| --- | --- | --- | --- | --- | --- | --- | --- | --- |
| Subgroup | | Blood pressure level | DII Quantile 0.25 | | DII Quantile 0.5 | | DII Quantile 0.75 | |
|  |  |  | β (95% CI) | P | β (95% CI) | P | β (95% CI) | P |
| Gender | Male |  |  |  |  |  |  |  |
|  |  | Normal | Ref. | Ref. | Ref. | Ref. | Ref. | Ref. |
|  |  | High-normal  (with history of HTN) | 0.11(-1.37,1.59) | 0.883 | 0.41(-1.28,2.10) | 0.631 | 0.52(-1.56,2.61) | 0623 |
|  |  | High-normal  (without history of HTN) | -1.07(-2.93,0.79) | 0.261 | -0.47(-2.79,1.84) | 0.689 | -0.26(-3.07,2.55) | 0.856 |
|  |  | New-Onset HTN | -0.18(-1.74,1.38) | 0.819 | -0.24(-2.43,1.95) | 0.829 | 1.80(-0.90,4.51) | 0.192 |
|  |  | HTN | 0.38(-1.55,2.32) | 0.670 | 0.52(-1.63,2.66) | 0.639 | 1.35(-1.86,4.55) | 0.410 |
|  | Female |  |  |  |  |  |  |  |
|  |  | Normal | Ref. | Ref. | Ref. | Ref. | Ref. | Ref. |
|  |  | High-normal  (with history of HTN) | 0.09(-0.72,0.90) | 0.824 | -0.36(-1.33,0.62) | 0.475 | -0.25(-1.70,1.19) | 0.729 |
|  |  | High-normal  (without history of HTN) | 0.14(-1.95,2.22) | 0.898 | -0.10(-1.58,1.39) | 0.897 | -0.77(-3.94,2.40) | 0.634 |
|  |  | New-Onset HTN | -0.52(-1.41,0.37) | 0.252 | **-1.38-**(**2.56,-0.20**) | **0.022** | -1.73(-4.01,0.56) | 0.134 |
|  |  | HTN | -0.01(-0.95,0.92) | 0.976 | -1.28(-2.67,0.11) | 0.072 | -0.73(-2.90,1.45) | 0.512 |
| Age(years) | 18~ |  |  |  |  |  |  |  |
|  |  | Normal | Ref. | Ref. | Ref. | Ref. | Ref. | Ref. |
|  |  | High-normal  (with history of HTN) | 0.45(-1.53,2.42) | 0.659 | 0.46(-1.16,2.07) | 0.580 | -0.11(-2.48,2.27) | 0.931 |
|  |  | High-normal  (without history of HTN) | - | - | - | - | - | - |
|  |  | New-Onset HTN | 0.39(-1.16,2.42) | 0.935 | 3.64(-9.51,16.78) | 0.589 | 5.46(-5.77,16.69) | 0.343 |
|  |  | HTN | 5.00(-13.09,33.10) | 0.589 | 13.53(-6.05,33.10) | 0.179 | 14.86(-3.04,32.76) | 0.107 |
|  | 35~ |  |  |  |  |  |  |  |
|  |  | Normal | Ref. | Ref. | Ref. | Ref. | Ref. | Ref. |
|  |  | High-normal  (with history of HTN) | 0.02(-1.01,1.06) | 0.963 | -0.92(-2.04,0.20) | 0.108 | 0.37(-1.27,2.01) | 0.658 |
|  |  | High-normal  (without history of HTN) | -1.35(-4.96,2.25) | 0.462 | -0.95(-4.70,2.80) | 0.621 | -1.04(-4.10,2.01) | 0.504 |
|  |  | New-Onset HTN | -0.28(-1.78,1.22) | 0.715 | -1.42(-3.32,0.49) | 0.147 | 0.74(-2.74,4.22) | 0.677 |
|  |  | HTN | -1.32(-3.31,0.66) | 0.192 | **-2.54(-4.76,-0.31)** | **0.026** | -2.91(-8.53,2.72) | 0.312 |
|  | 55~ |  |  |  |  |  |  |  |
|  |  | Normal | Ref. | Ref. | Ref. | Ref. | Ref. | Ref. |
|  |  | High-normal  (with history of HTN) | -1.07(-2.65,0.51) | 0.186 | -0.74(-2.64,1.16) | 0.448 | -1.85(-5.99,2.29) | 0.383 |
|  |  | High-normal  (without history of HTN) | -2.94(-4.73,-1.16) | 0.001 | -2.94(-6.33,0.46) | 0.091 | -1.99(-11.23,7.24) | 0.673 |
|  |  | New-Onset HTN | -2.12(-4.07,-0.17) | 0.034 | -1.13(-3.57,1.30) | 0.363 | -1.85(-6.23,2.52) | 0.407 |
|  |  | HTN | -1.07(-3.05,0.67) | 0.230 | -1.01(-3.05,1.04) | 0.335 | -1.89(-6.23,2.46) | 0.396 |
|  | 65~ |  |  |  |  |  |  |  |
|  |  | Normal | Ref. | Ref. | Ref. | Ref. | Ref. | Ref. |
|  |  | High-normal  (with history of HTN) | -0.95(-2.58,0.67) | 0.253 | 1.45(-0.59,3.48) | 0.164 | 2.79(-1.78,7.37) | 0.233 |
|  |  | High-normal  (without history of HTN) | 0.55(-1.17,2.28) | 0.530 | 1.66(-0.25,3.57) | 0.090 | 1.20(-3.38,5.79) | 0.608 |
|  |  | New-Onset HTN | -0.33(-1.80,1.15) | 0.666 | 0.40(-1.61,2.41) | 0.696 | 1.48(-3.10,6.07) | 0.527 |
|  |  | HTN | -0.52(-2.18,1.13) | 0.536 | 1.24(-1.03,3.51) | 0.285 | 3.09(-1.62,7.81) | 0.200 |
| Nation | Han |  |  |  |  |  |  |  |
|  |  | Normal | Ref. | Ref. | Ref. | Ref. | Ref. | Ref. |
|  |  | High-normal  (with history of HTN) | -0.17(-1.00,0.66) | 0.694 | -0.18(-1.04,0.68) | 0.690 | -0.27(-1.25,0.72) | 0.594 |
|  |  | High-normal  (without history of HTN) | -0.18(-1.73,1.37) | 0.823 | -0.27(-1.61,1.06) | 0.690 | -1.33(-3.27,0.61) | 0.179 |
|  |  | New-Onset HTN | -0.46(-1.45,0.53) | 0.362 | **-1.28(-2.22,-0.34)** | **0.008** | -0.75(-2.51,1.01) | 0.402 |
|  |  | HTN | 0.07(-1.06,1.19) | 0.905 | -0.37(-1.68,0.94) | 0.584 | -0.13(-1.75,1.48) | 0.870 |
|  | Minority |  |  |  |  |  |  |  |
|  |  | Normal | Ref. | Ref. | Ref. | Ref. | Ref. | Ref. |
|  |  | High-normal  (with history of HTN) | 0.56(-0.41,1.54) | 0.258 | -0.33(-2.53,1.86) | 0.767 | 0.66(-2.64,3.97) | 0.694 |
|  |  | High-normal  (without history of HTN) | -0.98(-3.42,1.46) | 0.432 | -0.77(-4.07,2.54) | 0.649 | -0.92(-5.87,4.08) | 0.717 |
|  |  | New-Onset HTN | -0.17(-1.53,1.20) | 0.812 | 0.32(-2.28,2.92) | 0.810 | 1.42(-3.86,6.70) | 0.599 |
|  |  | HTN | -0.03(-1.17,1.11) | 0.961 | -1.73(-4.22,0.77) | 0.177 | -0.64(-6.77,5.49) | 0.838 |
| Occupational physical activity intensity | Light |  |  |  |  |  |  |  |
|  |  | Normal | Ref. | Ref. | Ref. | Ref. | Ref. | Ref. |
|  |  | High-normal  (with history of HTN) | 0.08(-0.75,0.77) | 0.973 | -0.17(-0.88,1.03) | 0.873 | -0.75(-1.16,0.83) | 0.741 |
|  |  | High-normal  (without history of HTN) | -0.11(-1.38,2.03) | 0.710 | -0.33(-2.19,1.97) | 0.917 | -1.38(-3.74,3.08) | 0.850 |
|  |  | New-Onset HTN | -1.19(-1.30,0.41) | 0.311 | -0.76(-2.43,0.04) | 0.058 | -1.30(-2.46,0.95) | 0.384 |
|  |  | HTN | -0.14(-0.64,1.11) | 0.602 | -0.09(-1.59,1.31) | 0.851 | -0.64(-1.62,1.43) | 0.905 |
|  | Moderate |  |  |  |  |  |  |  |
|  |  | Normal | Ref. | Ref. | Ref. | Ref. | Ref. | Ref. |
|  |  | High-normal  (with history of HTN) | -0.72(-2.01,0.58) | 0.279 | -0.73(-2.28,0.82) | 0.360 | 0.90(-1.91,3,70) | 0.531 |
|  |  | High-normal  (without history of HTN) | -1.77(-3.66,0.12) | 0.068 | -1.07(-3.46,1.31) | 0.379 | -1.04(-4.05,1.96) | 0.496 |
|  |  | New-Onset HTN | -0.03(-1.86,1.81) | 0.978 | -0.09(-2.34,2.15) | 0.936 | 0.98(-2.69,4.65) | 0.601 |
|  |  | HTN | -0.47(-2.31,1.37) | 0.617 | -1.47(-3.47,0.54) | 0.152 | 0.40(-4.12,4.93) | 0.862 |
|  | Heavy |  |  |  |  |  |  |  |
|  |  | Normal | Ref. | Ref. | Ref. | Ref. | Ref. | Ref. |
|  |  | High-normal  (with history of HTN) | 0.77(-3.34,4.88) | 0.715 | -0.58(-5.71,4.56) | 0.826 | -2.51(-8.09,3.07) | 0.381 |
|  |  | High-normal  (without history of HTN) | 2.55(-1.75,6.84) | 0.249 | -0.18(-5.31,4.95) | 0.946 | -3.47(-9.47,2.53) | 0.260 |
|  |  | New-Onset HTN | -0.28(-4.73,4.17) | 0.902 | -1.39(-6.87,4.08) | 0.619 | -4.51(-11.20,2.18) | 0.190 |
|  |  | HTN | -0.91(-5.85,4.03) | 0.718 | -3.19(-23.46,17.09) | 0.759 | -2.37(-71.08,66.33) | 0.946 |
